# Research on Acute Toxicity and Pharmacodynamics of Jinyulian Oral Solution

**DOI:** 10.1007/s12013-015-0604-x

**Published:** 2015-05-29

**Authors:** Wan-Qing Cheng, Zhen-Jian Zhang, Chuan-Bin Cao, Xiao-Wen Ye, Wang-Ping Pan, Si-Ying Ye, Bo Zhu

**Affiliations:** 1 Department of Pharmacy, Suizhou Hospital, Hubei University of Medicine, Suizhou, 441300 Hubei Province China; 2 Microbial Chamber, The Hubei Provincial Food and Drug Supervision and Inspection Institute, Wuhan, 430064 Hubei Province China; 3 The Teaching and Research Group of Microbiology, Tongji Medical College, HUST, Wuhan, 430030 Hubei Province China

**Keywords:** Jinyulian Oral Solution, Acute toxicity, Pharmacodynamics

## Abstract

A research on Jinyulian Oral Solution was conducted and the objectives were to discover its possible acute toxicity and antibacterial effects when used in vitro and in vivo. Regarding the acute toxicity test, Kunming mice were fed a maximum amount of the solution as their stomachs could hold, i.e., 40 mL kg^−1^. To ascertain the minimum inhibitory concentration (MIC) of the solution, two types of germs, i.e., *Staphylococcus aureus* and *Escherichia coli*, were selected and tube dilution method was adopted. An antibacterial experimental model relying on animals’ body was developed for the researchers to observe the solution’s antibacterial effects. Test results showed that no abnormalities were discovered within 14 days after the initial date of testing and the mice grew as normal when fed with an amount of the solution 250 times of a normal clinical doze (In this case a man was assumed to weigh 60 kg.) and that the solution demonstrated obvious antibacterial effects on the two types of selected germs. The respective measured MIC_50_ and MIC_90_ values of the two germs were 3.2, 12.8, 6.4, and 25.6 mg L^−1^. Therefore, it is reasonable to conclude that Jinyulian Oral Solution possesses no acute toxicity but obvious antibacterial effects on the two before-mentioned germs.

## Introduction

Jinyulian Oral Solution, a decoction made of herba houttuyniae, fructus forsythia, radix isatidis, *Honeysuckle*, and liquorice, etc. [4], has been prescribed by Suizhou Central Hospital for patients suffered from acute or chronic pharyngitis, and tonsillitis [2] for years. To understand its working mechanism and assess its effects objectively, a research that focused on its acute toxicity and the antibacterial effects when used in vitro and in vivo was carried out, the details of which are reported as below.

## Testing Materials

*Medicines* used include Jinyulian Oral Solution (manufactured by the Solution Production Center of Suizhou Central Hospital; Certified Number: 070308; Specification: 10 mL per bottle) and Gentamicin sulfate injection (manufactured by Hubei Tianyao Pharmaceutical Limited; Certified Number: 20060502; Specification: 80,000 U mL^−1^).

*Bacterial Strains* Twenty bacterial strains, 10 containing *Staphylococcus aureus*, and the other 10 containing *Escherichia coli*, were isolated by the Department of Gynaecology and the Urologic Dept. of Tongji Hospital from their laboratories. All the bacterial strains were tested by Gram-stained microscopy, coagulase butter, and VITEK microbial analyzer. Two bacterial strains coded ATCC 25923 and 25922 containing *S. aureus* and *E. coli*, which were acquired from the clinical laboratory of Tongji Hospital, were regarded as the representative strains to assess the antibacterial activity of the solution.

*Animals* used for testing were mice in SPF grade weighing about 20–22 g; half of them were male and the other half female. They were provided by Hubei Center of Laboratory Animals. The animal breeding license and the license for animals in SPF grade to be used in laboratory experiments bear the numbers of SCXK (Hubei Province) 2003–0005 and SYXK (Hubei Province) 2003–0009, respectively.

*Reagents* used for the testing include MH broth (produced by American Difo Laboratory, Detroit, USA), Tweert-20 (produced by Shanghai Biochemical Reagent Co., Ltd.), sheep blood (furnished by the Laboratory Animals Center of Tongji Medical College, HUST), and human blood (supplied by Tongji Hospital’s Blood Bank, HUST).

## Testing Methods

### Acute Toxicity Test

Based on preliminary test results, 20 mice as described before were selected for the test, 10 male and 10 female. Within 12 h before the testing commenced, the mice were prohibited from feeding themselves but were allowed to drink water. When the testing time was due, mice were weighed and fed once in the morning and once in the afternoon with Jinyulian Oral Solution into their stomachs, 40 mL kg^−1^ each. After the feed, the mice’s behavior was observed immediately. On the 1st day mice were observed three times a day and after that observations were made once a day until the 14th day elapsed. During observation, details of mice were recorded including their skin, mucous membrane, changes in fur color, eyes, respiration, circulation, central nervous system (CNS), and conscious behavior. When the testing was over, mice were weighed and dissected for autopsy.

### In Vitro Antibacterial Test of the Solution [8]

#### Isolation, Culturing, and Assessment of Bacterial Strains

Suspectable specimens were implanted onto the blood agar decks, which were placed into a 35 °C couveuse containing 5 % carbon dioxide for about 18–24 h before observations were made. Bacterial strains isolated were firstly assessed through Gram-stained microscopy, coagulase butter, and VITEK microbial analyzer (NHI and GNI), and then they were reserved at temperatures of 4 °C and −70 °C in semisolid culture-medium added with glycerol for future use.

#### Method for Antibacterial Testing

##### Preparation of Antibacterial Stock Solution

The mass concentration of commonly used stock solution was 5120 mg kg^−1^, and it was more than ten times the concentration of solution to be measured. With regard to measuring the MIC, provisions offered by National Committee for Clinical Laboratory Standards 1999 (NCCLS) [7] were referred to and two-fold tube dilution method was adopted. Two bacterial strains with one containing *S. aureus* and the other containing *E. coli*, coded ATCC 259223 and 25922, respectively, were the strains under quality control for monitoring the whole experiment process.

##### Procedures for Diluting the Antibacterial Solution

Sixteen aseptic tubes in 8 × 10 mL size were placed in two rows, eight tubes in each row. Another three aseptic tubes were arranged as references with each containing broth, germs, and air, respectively. Two milliliter of MH broth was added to the tubes with the exception of the 1st tube in both rows. Then 2 mL Jinyulian Oral Solution was added to the first and the second tube in both rows. Then the mixture in the four tubes were diluted with MH broth to the highest concentration to be measured. And then, 2 mL mixture was sucked out from the 2nd tube and injected into the 3rd tube. When the mixture in the 3rd tube was properly mixed by shaking, 2 mL admixture was sucked out, and disposed of. The before-mentioned process was repeated until the mixture in the 8th tube was diluted. In the end, the mass concentrations of Jinyulian Oral Solution for each tube came to 25.6, 12.8, 6.4, 3.2, 1.6, 0.8, 0.4, and 0.2 mg L^−1^ in a sequential order.

##### Preparation of Inoculums and Inoculation

About 3–5 newly grown fresh bacterial strains, which were under quality control, were chosen and inoculated into about the 3–5 mL MH broth. After the inoculums were bred at 35 °C for 5 h by shaking, their concentrations were adjusted to 0.5 McIntosh turbidimetric tube by physiological saline and were diluted to 1/10 (containing about 10^7^ germs) with MH broth. The mixture was inoculated within 15 min after dilution [5].

Regarding the inoculation, firstly 0.1 mL bacterial detection solution was borrowed through pipettes, and then it was added to tubes in the 1st row following an ascending concentration. Standard bacterium were added to tubes in the 2nd row. When doing so, the sucker of injectors shall be inserted below the liquid level and its contacts with the inner surface of tubes should be avoided. Vibration of tubes should be prevented after bacterial solution is injected.

##### Incubation

To prevent evaporation, tubes were placed in a 35 °C incubator for about 16–20 h. Records were made on the concentration ranges in which growth of *S. aureus* and *E. coli* at MIC_50_ and MIC_90_ was stopped.

### In Vivo Antibacterial Effects of the Solution [1]

#### Preliminary Tests

Twelve Kunming mice were classified into four groups at random, with three mice in each group. The bacterial strains to be isolated from laboratory tests were bred by the Antibiotics Division of Hubei Institute for Food and Drug Control. After 2 days, the broth containing germs was injected into the mice. The four groups of mice were named “intravenous inoculation group of *S. aureus*,” “the intraperitoneal inoculation group,” “intravenous inoculation group of colibacillus,” and “the intraperitoneal inoculation group,” respectively. Test results showed that three mice in the intravenous inoculation group of colibacillus died on the 2nd day and that the remaining mice in three groups did not demonstrate any abnormalities or suffered death even after the 4th day of the experiment. Thus, intravenous inoculation of colibacillus was selected for future tests.

Twelve Kunming mice were classified into four groups at random, with three mice in each group. Broth containing colibacillus was the testing material. Each mouse of the 1st group was injected 0.6 mL broth, the 2nd group 0.6 mL, the 3rd group 0.2 mL, and the 4th group 0.1 mL. Results are that one mouse of the 1st group died 4 h after the test and the remaining eight mice of the first three groups died on the 2nd day. Regarding the mice of the 4th group, one died on the morning of the 2nd day and the other two mice died in the afternoon. Therefore, broth containing colibacillus, the ratio of which to physiological saline was 1–8, would be injected intraperitoneally into each mouse at a doze of 0.4 mL.

#### Formal Tests

Fifty Kunming mice were classified into five groups at random, with 10 mice in each group. The five groups were named “the group to be given a large doze (Jinyulian Oral Solution, undiluted, 0.4 mL 20 g^−1^),” “the group to be given a small doze (Jinyulian Oral Solution, diluted by 50 %, 0.4 mL 20 g^−1^),” “the positive group (Gentamicin 2000 U 20 g^−1^, 0.4 mL 20 g^−1^),” “the modeling group,” and “the blank control group” (Mice in which group were injected the same doze of physiological saline). With the exception of mice in the blank control group, each mouse in the remaining groups was injected 0.4 mL broth containing colibacillus while they were fed with the solution. It continued even after the inoculation was done. In the process, details concerned with the death of mice were observed and recorded. After 1 week, the mice were examined by *χ* ^2^.

## Research Findings

### Acute Toxicity Test

When Jinyulian Oral Solution was fed at a maximum doze of 40 mL kg^−1^, equivalent to the amount of 250 times of a clinical doze for a man weighing 60 kg, into the stomachs of mice, no abnormalities were observed as far as mice’s skin, mucous membrane, color, eyes, respiration, CNS, and conscious behavior were concerned and the mice gained weight. At the end of the experiment, mice were killed and no obvious pathological changes in their organs were visible to naked human eyes.

### In Vitro Antibacterial Effects of Jinyulian Oral Solution

Jinyulian Oral Solution produces in vitro antibacterial effects against *S. aureus* and *E. coli*. The MIC_50_ and MIC_90_ of both germs are 3.2, 12.8, 6.4, and 25.6 mg L^−1^ respectively. For details, please refer to Tables 1 and 2 as below.

**Table 1:** In vitro antibacterial activity of Jinyulian Oral Solution for *Staphylococcus* (mg sL^−1^)

Germ	Range	MIC_50_	MIC_90_
*Staphylococcus* ATCC25923	–	3.2	3.2
Isolated strains with *Staphylococcus* (10)	0.8–12.8	6.4	12.8

**Table 2:** In vitro antibacterial activity of Jinyulian Oral Solution for *E. coli* (mg L^−1^)

Germ	Range	MIC_50_	MIC_90_
*E. coli* ATCC25923	–	6.4	6.4
E. coli (10)	1.6–25.6	6.4	25.6

### Regarding the In Vivo Antibacterial Effects of the Jinyulian Oral Solution, Please Refer to Table 3

## Further Discussion

Jinyulian Oral Solution, which has been prescribed by doctors in the E.R.T. Dept. and the T.E.M. Dept. of Suzhou Central Hospital for years, is the effective medicine for patients inflicted by acute and chronic pharyngitis and tonsillitis. With the technological advancement in making decoction in recent years, herbal extracts have been used to replace raw medicinal herbs [6], ensuring adequate extraction of effective elements and the clarity of the solution. Besides, production efficiency has been improved and the time required for manufacturing reduced, significantly improving its effectiveness. Preparations to be taken orally will also be developed. Stability of Jinyulian oral solution were conform to the rules by several test methods including high light test, high temperature test, high humidity test, accelerated test, room temperature sample observing [3]. To date, the solution has been prescribed to 973 patients infected with acute pharyngitis and 1287 patients suffering from chronic pharyngitis. Of them, 1789 patients have been cured of, 328 patients’ condition has changed for the better, and 143 patients do not show any betterment. The effectiveness ratio comes to 93.7 %.

**Table 3:** Protective effect of Jinyulian Oral Solution for the mice infected with *E. coli* (*n* = 10)

Group	Doze	Number of Mice Died
Large doze group	20 mL L^−1^	6^a^
Low doze group	10 mL L^−1^	8
Positive group	40,000 U kg^−1^	0^b^
Modeling group	20 mL kg^−1^	10
Blank control group	20 mL kg^−1^	0^b^

A comparison with the model was made, and ^a^ *P* < 0.05, ^b^ *P* < 0.01

Regarding the two main ingredients of Jinyulian Oral Solution, both forsythia suspensa Vahl and cordate houttuynia help clear a patient’s heat, detoxicate, and reduce swelling. Their difference is that forsythia suspensa Vahl may dissipate binds and cordate houttuynia may help a patient discharge purulence. When the two main ingredients are mixed with *Honeysuckle*, radix isatidis, and liquorice, the medicine has pleasant effects to clear away heat, detoxicate, reduce swelling, and soothe a patient’s lung and curb coughs.

The acute toxicity test of Jinyulian Oral Solution demonstrates that no abnormalities were observed when mice were fed a maximum doze of 40 mL kg^−1^ solution, equivalent to 250 times of a clinical doze for a man weighing 60 kg, into their stomachs. It is justified that toxicity of the solution is minimal.

With regard to the in vitro antibacterial test, bacteriological tests were carried out through the tube dilution method and results showed that Jinyulian Oral Solution may produce antibacterial effects against *S. aureus* and *E. coli*. Also, the antibacterial activity of the solution was assessed in the research. In vivo antibacterial testing exhibited that Jinyulian Oral Solution may produce protective effects on mice infected with *E. coli*.
